# Nuclear expression of TCF4/TCF7L2 is correlated with poor prognosis in patients with esophageal squamous cell carcinoma

**DOI:** 10.1186/s11658-016-0006-0

**Published:** 2016-07-28

**Authors:** Hideyuki Ishiguro, Takehiro Wakasugi, Yukio Terashita, Nobuhiro Sakamoto, Tatsuya Tanaka, Hiroyuki Sagawa, Tomotaka Okubo, Hiromitsu Takeyama

**Affiliations:** Department of Gastroenterological Surgery, Nagoya City University Graduate School of Medical Science, 1 Kawasumi, Mizuho-cho, Mizuho-ku, Nagoya, 467-8601 Japan

**Keywords:** Esophageal cancer, Immunohistochemistry, Prognosis, TCF4/TCF7L2, Wnt signal, Surgery, Clinicopathological factor

## Abstract

The prognosis for patients with esophageal cancer remains poor. Therefore, the identification of novel target molecules for the treatment of esophageal cancer is necessary. Here, we investigated the clinicopathological significance of transcription factor 4/transcription factor 7-like 2 (TCF4/TCF7L2) in resectable esophageal squamous cell carcinoma (ESCC), because TCF4/TCF7L2 expression has not been studied in esophageal cancer previously.

This study included 79 patients with esophageal cancer who underwent surgery between 1998 and 2005. The expression of the TCF4/TCF7L2 protein in the nucleus of esophageal cancer cells was analyzed using immunohistochemistry. We examined the correlation between TCF4/TCF7L2 expression, clinicopathological factors, and prognosis in patients with ESCC.

TCF4/TCF7L2 was expressed in 57 % (45/79) of patients. TCF4/TCF7L2 expression was correlated with T factor (T1 vs. T2-4, *p* = 0.001), stage (I vs. II-IV, *p* =0.0058), Ly factor (*p* =0.038), and V factor (*p* =0.038) and did not correlate with age, gender or N factor. Furthermore, patients who were positive for TCF4/TCF7L2 had a significantly lower survival rate than those who were negative for TCF4/TCF7L2 (log-rank test, *p* = 0.0040). TCF4/TCF7L2 expression significantly affected the survival of patients with ESCC. Positive expression of TCF4/TCF7L2 was correlated with a poor prognosis after a curative operation in patients with ESCC.

## Introduction

The prognosis of patients with esophageal cancer remains poor, emphasizing the need for the development of new treatment strategies. Today, the overall 5-year survival rate is less than 50 %, despite the use of multimodal therapies. Even in early-stage disease, many patients develop a local recurrence of tumors or distant metastasis within a short period of time after operation. To develop novel treatment strategies, it is important to understand the biological behavior of esophageal cancer. Recent studies identified several genes and molecules involved in the origin and/or progression of esophageal cancer, including TP53 [1], *deleted in esophageal cancer 1* (*DEC1*) [2], *deleted in colorectal cancer* (*DCC*) [3], *deleted in lung cancer 1* (*DLC1*) [4], cyclin D1 [5], adenomatous polyposis coli (APC) [6], and *survivin* [7]. However, the precise mechanisms that underlie the development and progression of esophageal squamous cell carcinoma (ESCC) remain unclear.

The Wnt signaling pathway regulates important cellular processes, including development and differentiation, apoptosis, immunologic and inflammatory responses, cell-cycle progression and cellular division [8, 9]. Transcription factor 4/transcription factor 7-like 2 (TCF4/TCF7L2) is a key molecule of the Wnt signaling pathway, which acts as a transcriptional factor in the nucleus [8, 10]. Downstream genes of the Wnt signaling pathway include cyclin D1 and c-myc. To the best of our knowledge, no reports have described the clinicopathological significance of TCF4/TCF7L2 protein expression in the progression of various malignancies.

In this study, we investigated the clinicopathological significance of TCF4/TCF7L2 protein expression in 79 patients with resectable ESCC.

## Materials and methods

### Tissue samples

Samples were obtained from 79 patients with ESCC who underwent operation at the Department of Gastroenterological Surgery, Nagoya City University Medical School between 1998 and 2005 without pre-operative chemotherapy or radiation. The tumors were classified according to the guidelines for clinical and pathological studies on carcinoma of the esophagus. The samples were used after obtaining written consent from the patients.

### Immunohistochemistry

Immunohistochemical staining was performed on formalin-fixed, paraffin-embedded primary human ESCC tissues using the monoclonal anti-TCF4 antibody (Cell Signaling, NY) at 1:200. Paraffin-embedded tumor sections were deparaffinized, rehydrated, heat-treated by microwaving in 10 mM citrate buffer for 15 min for antigen retrieval, and cooled to room temperature. The sections were then treated with 0.3 % H_2_O_2_ in methanol for 30 min to neutralize the endogenous peroxidases, blocked with nonspecific goat serum for 10 min, and incubated with the H-100 antibody overnight at room temperature in a humid chamber. The immunoreactive protein was detected with a DAKO Envision System, HRP (DAB), and sections were counterstained with hematoxylin. Two independent investigators subjectively assessed the immunostaining of TCF4, and discordant results were resolved by consultation with a third investigator. For the evaluation of TCF4 expression, immunostaining was considered positive only when unequivocally strong nuclear staining was present in more than 50 % of the tumor cells, as analyzed using a light microscope. Cases with faint staining only were considered negative.

### Statistical analysis

The chi-squared test was used to analyze the correlations between the clinicopathological factors and the expression of TCF4/TCF7L2. The survival rates were calculated according to the Kaplan-Meier method. Multivariate analysis of Cox’s proportional hazard risk model was used to obtain the conditional risk of death due to ESCC. Differences were considered statistically significant for *P* values less than 0.05.

## Results

### Expression of TCF4/TCF7L2 in ESCC

**Fig. 1:**
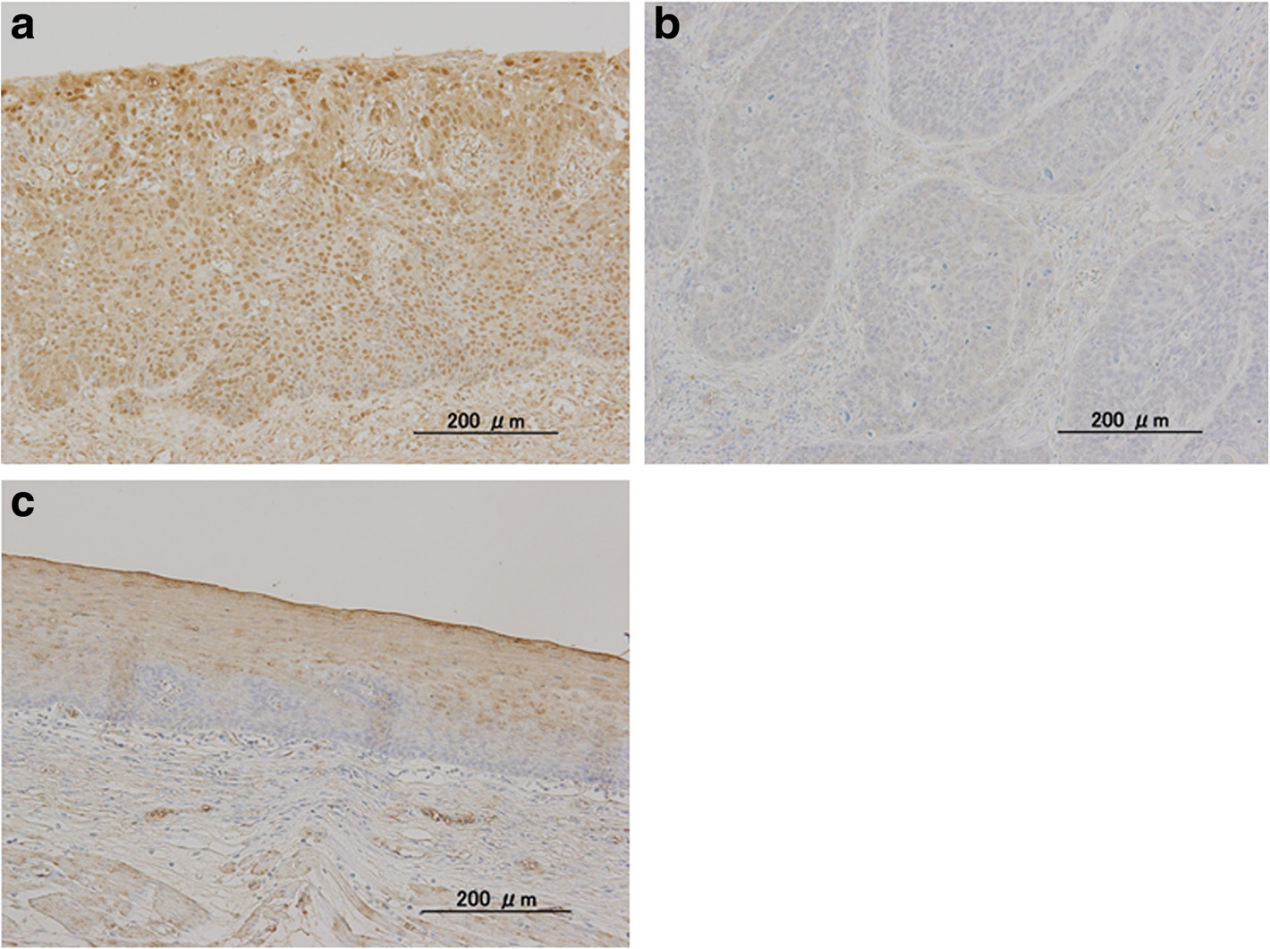
Representative immunostaining for TCF4/TCF7L2. **a** – Positive staining for TCF4/TCF7L2 in tumor cells. **b** – Negative staining for TCF4/TCF7L2 in tumor cells. **c** – Representative immunostaining for TCF4/TCF7L2 in normal esophageal mucosa

First, we investigated the expression of the TCF4/TCF7L2 protein in ESCC tissues using immunohistochemistry. Representative images of TCF4/TCF7L2 immunostaining are shown in Fig. 1. Typical ESCC cells showed diffuse nuclear staining for TCF4/TCF7L2, and the cell membrane and cytoplasm showed little to no staining. There is almost no nuclear staining in normal esophageal mucosa of resected tissue (Fig. 1c). Immunostaining for TCF4/TCF7L2 was positive in 56.9 % (45/79) of patients. TCF4/TCF7L2 expression correlated significantly with the T factor, p-stage, lymphatic invasion and vein invasion and did not correlate with the N factor (Table 1).

**Table 1:** Correlation of TCF4 IHC in esophageal cancer with clinicopathological factors, including patient and tumor characteristics

		No. of patients (*n* = 79)		
Characteristics	Case	TCF4(+)	TCF4(−)	*p*-value
Age at surgery				
<65 years	44	25	19	
>65 years	35	20	15	0.614
Gender				
Male	63	35	28	
Female	16	10	6	0.573
Tumor status				
T1	15	3	12	
T2	9	5	4	
T3	32	19	13	
T4	22	17	5	
T1 vs T2-4				0.001
Lymph node status				
N0	18	7	11	
N1	61	38	23	
N0 vs N1				0.078
Pathological stage				
I	11	2	9	
II	13	9	4	
III	22	10	13	
IV	32	23	9	
I vs II-IV				0.0058
Lymphatic invasion				
Negative	15	5	5/8	
Positive	47	30	22/41	
Unknown	16			0.038
Blood vessel invasion				
Negative	28	11	13/21	
Positive	34	24	14/28	
Unknown	7			0.038

### Survival curves and expression of TCF4/TCF7L2

**Fig. 2:**
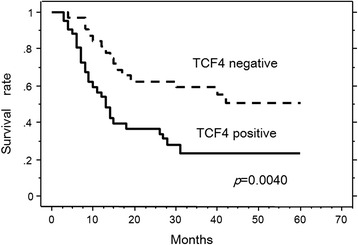
Kaplan-Meier survival curve for esophageal cancer patients, classified as either positive or negative for TCF4/TCF7L2 immunostaining. TCF4/TCF7L2 status is strongly associated (log-rank, *p* = 0.0040) with patient survival

Next, we investigated the correlation between immunostaining for TCF4/TCF7L2 and survival of patients with ESCC after surgery. TCF4/TCF7L2 had a significant effect on patient survival (Fig. 2), and patients with positive staining for TCF4/TCF7L2 had significantly shorter survival after surgery than patients with negative staining (16.7 ± 1.7 months [*n* = 45] vs. 30.6 ± 2.6 months [*n* = 34], respectively; *p* = 0.004 by log-rank test; Fig. 2).

Univariate analysis showed that, among the clinicopathological factors examined in this study, the extent of primary tumor (risk ratio, 4.184; *p* < 0.0001), lymph node metastasis (risk ratio, 4.149; *p* < 0.0001), lymphatic invasion (risk ratio, 6.622; *p* = 0.003), vein invasion (risk ratio, 2.816; *p* = 0.0003), and immunostaining for TCF4/TCF7L2 (risk ratio, 2.506; *p* = 0.0049) were statistically significant prognostic factors. Multivariate analysis revealed that TCF4/TCF7L2 expression was not an independent prognostic factor (data not shown).

## Discussion

The Wnt signaling pathway plays important roles in axis formation during early vertebrate development [11]. Upon Wnt signaling, the phosphorylation of beta-catenin is suppressed through undefined mechanisms, and beta-catenin functions as a transcriptional regulator in the nucleus together with TCF4/LEF1 [12, 13]. A number of downstream genes, such as c-myc [12], cyclin D1 [14, 15], c-jun, fra-1, uPAR, ZO-1 [16], and novel protein band 4.1 like 4 *(NBL4)* [17] have been reported; however, the precise regulatory mechanisms remain to be resolved. Alterations of APC, AXIN, or beta-catenin itself, lead to the accumulation of beta-catenin in the cytoplasm and/or nucleus, resulting in the unregulated transcription of downstream genes [12–14, 16–18]. However, in esophageal cancer cells, the frequency of beta-catenin accumulation in the nucleus is lower than in colon and liver cancer [19]

TCF4/TCF7L2 is a major component of the Wnt signaling pathway. However, because few reports have described the mechanisms mediating Wnt signaling activation in ESCC, the factors that regulate TCF4/TCF7L2 expression in this type of cancer are not known.

In many cancer cells, TCF4/TCF7L2 is localized to the nucleus [20]. Consistent with this observation, our current experiments show that TCF4/TCF7L2 is also expressed in the nucleus of ESCC cells (Fig. 1). In colon cancer cells, TCF4/TCF7L2 is located in the nucleus with beta-catenin [20]. By contrast, for esophageal squamous cancer cells, our data suggest that TCF4/TCF7L2 alone is located in the nucleus without beta-catenin, because beta-catenin is not detected in the nucleus in ESCC [19]. However, the mechanisms that regulate TCF4/TCF7L2 expression in ESCC remain unclear.

There are a few reports that the Wnt signaling pathway is activated in ESCC. Cyclin D1, a downstream gene of the Wnt signaling pathway, is highly expressed in ESCC [21, 22]. Other mechanisms of translocation to the nucleus for TCF4/TCF7L2 may exist. Downstream genes of the Wnt signal pathway in esophageal cancer may be activated by TCF4/TCF7L2 activation.

Because TCF4/TCF7L2 plays a role in cancer proliferation, additional studies are necessary to determine whether TCF4/TCF7L2 contributes to the growth of esophageal cancers. In our study, TCF4/TCF7L2 was correlated with the T factor of patients with ESCC (Table 1).

Interestingly, we found that TCF4/TCF7L2 predicted the prognosis of patients with ESCC. Therefore, our data suggest that TCF4/TCF7L2 is involved in the cell proliferation of esophageal carcinoma and that TCF4/TCF7L2 is a useful biomarker for predicting prognosis in patients with ESCC.

Several clinical studies have reported that TCF4/TCF7L2 is an indicator of poor prognosis or malignant potential in hepatocellular carcinomas [17] and colon cancer [11]. The current study may be the first report demonstrating that TCF4/TCF7L2 is correlated with the prognosis of patients with esophageal squamous cell carcinoma.

In this study, we found that increased expression of TCF4/TCF7L2 in the nucleus of cancer tissue cells was accompanied by the local progression of esophageal cancer (Fig. 1 and Table 2). In addition, patients with high nuclear expression of TCF4/TCF7L2 had a poorer prognosis (Fig. 2).

**Table 2:** Univariate analysis

Parameter	Risk ratio	95 % CI	*p*-value
Age at surgery			
<65 years	1		
>65 years	1.033	0.675-1.580	0.8814
Gender			
Female	1		
Male	1.057	0.628-1.777	0.8353
Primary tumor			
T1-3	1		
T4	4.184	2.610-6.711	<0.0001
Lymph node metastasis			
N0	1		
N1	4.149	2.242-7.692	<0.0001
Lymphatic invasion			
Negative	1		
Positive	6.622	2.398-18.18	0.003
Vein invasion			
Negative	1		
Positive	2.816	1.597-4.975	0.0003
Immunostaining for TCF4			
negative	1		
positive	2.506	1.321-4.739	0.0049

CI, confidence interval

Additionally, whether *TCF4/TCF7L2* expression is mediated by other mechanisms will be the focus of future studies.

In patients with esophageal cancer, many prognostic markers, including cyclin D1, E-cadherin, and MDM2, have been reported [21, 23]. Furthermore, we also reported that pituitary tumor transforming gene 1 (*PTTG1*) [24], DNA fragmentation factor 45 (*DFF45*) [25], *NOTCH1* [15], VEGF-C [16], and DROSHA [26] may be prognostic markers of ESCC*.* Therefore, TCF4/TCF7L2 represents an additional potential prognostic indicator for patients with ESCC.

Although the precise molecular mechanisms through which TCF4/TCF7L2 is activated must be clarified, our data clearly indicate that TCF4/TCF7L2 may be a molecular target for the development of effective therapeutic agents for patients with esophageal cancer.

## Abbreviations

APC, adenomatous polyposis coli, DCC, deleted in colorectal cancer, DEC1, deleted in esophageal cancer 1, DFF45, DNA fragmentation factor 45, DLC1, deleted in lung cancer 1, ESCC, esophageal squamous cell carcinoma, NBL4, novel protein band 4.1 like 4, PTTG1, pituitary tumor transforming gene 1, TCF4/TCF7L2, transcription factor 4/transcription factor 7-like 2
